# The integrated effects of leader–member exchange social comparison on job performance and OCB in the Chinese context

**DOI:** 10.3389/fpsyg.2023.1094509

**Published:** 2023-01-19

**Authors:** Chunjiang Yang, Yashuo Chen, Aobo Chen, Syed Jameel Ahmed

**Affiliations:** ^1^School of Economics and Management, Northwest University, Xi’an, China; ^2^Sun Yat-sen Business School, Sun Yat-sen University, Guangzhou, China; ^3^School of Economics and Management, Yanshan University, Qinhuangdao, China; ^4^Department of Commerce, University of Balochistan, Quetta, Pakistan

**Keywords:** leader–member exchange social comparison, guanxi, Zhongyong thinking, job performance, organizational citizenship behavior

## Abstract

Although it has been long recognized that leader–member exchange social comparison (LMXSC) has critical implications for employee productivity, little attention has been given to systematically exploring the effects of LMXSC on employee performance in a specific cultural context. Integrating social exchange theory with social comparison theory, we examine a dual process model to explain how and when LMXSC affects employee performance outcomes in the Chinese context. Results based on multiphase, multisource data from China revealed that the mediating roles of employees’ perceived obligation toward the leader and self-esteem are examined simultaneously in the relationship between LMXSC and job performance and organizational citizenship behavior (OCB). Additionally, guanxi strengthens the connection between LMXSC and perceived obligation, while Zhongyong thinking erodes the connection between LMXSC and self-esteem. Taken together, these findings enhance our understanding of LMXSC in China.

## 1. Introduction

In the workplace, we seem unable to stop comparing ourselves to peers in various aspects, such as performance, salary (Kim et al., 2018), stress, health (Yang et al., 2021), status (Reh et al., 2022), and especially the leader-employee relationship. Without a doubt, leaders hold the keys to the huge resources that determine whether employees successfully complete their tasks and advance in their careers, and their access to these resources is largely contingent on their relationship with the leader (Moser et al., 2022). According to a survey in 2022, a startling 79% of employees will quit after obtaining insufficient appreciation from their superiors or establishing positive work relationships with them (Apollo Technical, 2022). Consequently, leader–member exchange social comparison (LMXSC) defined by Vidyarthi et al. (2010) as employees’ evaluations of their own leader–member exchange (LMX) relative to that of their peers has been one of the most important topics in the leader–employee relationship field over the years. LMXSC acknowledges that each leader-follower dyadic relationship is nested inside multiple leader-follower relationships (Martin et al., 2018), and that the comparison in terms of LMXs across employees significantly leads to their attitudinal and behavioral outcomes. To get a better understanding of the current of LMXSC research, we conducted a systematic review of the LMXSC literature and identified 23 relevant studies, as shown in Table 1. The existing research suggests that LMXSC plays a vital role in explaining, for example, employees’ job performance, organizational citizenship behavior (OCB), knowledge hiding behavior, and organizational commitment (Weng et al., 2020; Jahantab et al., 2021; Afshan et al., 2022). Although it has been known for a long time that LMXSC has significant consequences for employee productivity, most studies rely on only one theoretical perspective to understand its influences (Korman et al., 2020; Lapointe et al., 2020; Liu et al., 2022), which are largely limited to our systematically examining why and how LMXSC shapes employee job performance and OCB (Harris et al., 2014). In addition, most research on LMXSC have been conducted in western context (Lapointe et al., 2020), leaving it unclear if LMXSC has a comparable role in predicting the organizational behaviors of Chinese employees. Cultural factors may drive employees from diverse cultural backgrounds to adopt varying opinions towards LMXSC and thus moderate the relationship between LMXSC and outcomes (Rockstuhl et al., 2012). Thus, findings originating from western contexts may not necessarily be applicable to other cultural situations, or further findings may be uncovered in an eastern cultural context.

**Table 1:** Existing research on LMXSC as an antecedent.

Authors (year)	Samples	Theories	Mediators	Moderators	Outcomes
Vidyarthi et al. (2010)	India	/	/	/	Job performance; OCB
Tse et al. (2013)	China	Balance theory	A’s Contempt for B	Social comparison orientation; Coworker A’s LMX	Perception of help
Chan (2015)	/	Social influence theory	/	Politically skill	Ingratiation
Huang et al. (2015)	China	/	Procedural justice; Interpersonal justice	Organizational embodiment	Organizational deviance; Supervisor-directed deviance
Ott-Holland (2015)	United States	Attribution theory; Core affect theory	/	Interpersonal justice; Locus of causality for relationship building with one’s supervisor	Positive emotion; Negative emotion
Matta (2016)	United States	Social comparison emotions	/	Self-other overlap	Emotions
Vidyarthi et al. (2016)	India	Social comparison theory	/	Team orientation; Task interdependence.	Performance
Kim H. L. R. et al. (2017)	/	Impression management research	Coworker exchange	LMX; Equity sensitivity	Interpersonal citizenship behavior
Valdiviezo (2017)	United States	Social comparison theory; Attachment theory	Job embeddedness	/	Psychological ownership; Job satisfaction
Tse et al. (2018)	United States	Social comparison theory	Hostility toward to coworker	Procedural justice climate	Harmful behavior toward the coworker
Arain et al. (2019)	Kingdom of Saudi Arabia	/	/	/	Promotive voice; Prohibitive voice
Lee et al. (2019)	United States; China	Social exchange theory	Felt obligation	Psychological entitlement; Job performance	Job performance; Organizational commitment
Choi et al. (2020)	South Korea	The equality principles	Procedural justice climate; Relational conflict	/	Team performance; Individual performance
Flores et al. (2020)	/	Social cognitive theory; Social comparison theory	Self-efficacy	Ethical leadership	Job performance; OCB
Lapointe et al. (2020)	Canada	Social exchange theory	Organizational commitment	Perceived supervisor collective self-concept; Employee relational self-concept; Perceived supervisor relational self-concept	Task proficiency; Task adaptivity and proactivity
Korman et al. (2020)	United States	Social comparison theory	Hubristic pride	/	Coworker-direct social undermining
Sharma et al. (2020)	India	Social comparison theory	Envy	Aggression-preventive supervisor behavior	Uncivil behavior
Weng et al. (2020)	China	Social comparison theory	Envy toward coworkers	Cooperative goal interdependence; Competitive goal interdependence	Knowledge hiding behavior
Chen and Zhang (2021)	China	Group engagement model	Procedural justice	LMX; Group-level LMX differentiation	/
Pan et al. (2021)	China	Social comparison theory; EASI theory	Benign envy; Malicious envy	Perceived hubristic pride; Perceived authentic pride	Learning behavior; Social undermining
Shalendra and Kang (2021)	/	Social comparison theory	Employee-organization relationship	Supervisor’s organizational embodiment	Voice behavior
Lee et al. (2022)	United States	Social comparison theory	Psychological capital	/	Malicious envy; Benign envy
Liu et al. (2022)	China	Social identity theory	Perceived outsider status of the target	Trait self-control	Deviant behavior

To fill in these theoretical gaps in the current LMXSC research, we build an integrated theoretical framework for how and when LMXSC works in the Chinese context to affect employee outcomes. Specifically, we combine LMXSC research with the two most relevant theories, namely social exchange theory and social comparison theory. Social exchange theory asserts that employee performance is contingent upon reciprocal obligations stemming from the employee’s relationship with their leader (Cropanzano and Mitchell, 2005), whereas social comparison theory explains why social comparison processes within focal individuals in terms of leader-employee relationships are central to individual outcomes (Festinger, 1954). Both theories lay the foundation for the formulation and development of the LMXSC concept (Vidyarthi et al., 2010). This relevant and common theoretical foundation enables us to present a unified or integrated perspective for elucidating the process by which LMXSC translates into employee job performance and OCB.

Drawing upon social exchange theory, followers with good-quality LMX tend to obtain various resources provided by their leaders and thus have a strong motivation to improve their in-and out-role performance as a form of reciprocating their leaders’ payout (Erdogan and Liden, 2002). If employees have a high LMXSC, they seem to be more inclined to feel obligated to their leaders, which will affect their job performance and OCB (Hooper and Martin, 2008). Moreover, literature on social exchange posits that situational characteristics shape individuals’ feelings of obligation requested by the leader-employee relationship (Hollander, 1980). In China, the overlap between work and social relations is significantly more pervasive, and guanxi, an indigenous Chinese concept, describes “an informal, particularistic personal connection between two individuals who are bound by an implicit psychological contract to follow the social norm of guanxi” (Chen and Chen, 2004, p: 306). Supervisor-subordinate guanxi (hereinafter referred to as guanxi) specially refers to an employee’s personal, non-work relationships with a leader (Chen et al., 2013), which involve mutual commitment, loyalty, and trust. We argue that if employees with high LMXSC also have guanxi with their leaders, they will perceive an obligation to repay the resources from their leaders, thus strengthening the positive relationship between LMXSC and perceived obligation toward leaders.

On the other hand, drawing upon social comparison theory, individuals undertake social comparison processes in which they evaluate themselves by considering “information about one or more other people in relation to the self” (Wood, 1996, p: 520–521). Accordingly, those with a high LMXSC who compare themselves to their less fortunate coworkers are likely to create a positive self-image, while those with a low LMXSC tend to develop a negative self-image after comparing themselves to those in a better LMX position (Tse et al., 2018). As a fundamental self-image-related evaluation, self-esteem is defined as a person’s self-perception of his or her own value, worth, or competence (Pyszczynski et al., 2004). We argue that LMXSC has a positive relationship with employee self-esteem and leads to employees acting in a way congruent with their competence and value, such as by exhibiting high work performance and OCB. However, the positive effect of LMXSC on self-esteem may be mitigated if the employee has a high degree of Zhongyong thinking, a unique and significant mode of thinking in traditional Chinese culture (Wei et al., 2020). Zhongyong is the core content of Confucian thought, which has dominated Chinese society since ancient times and had a tremendous impact on the Chinese people (Yang et al., 2016). Zhong signifies center, the mean, and balance, neither leaning to one side nor the other, while Yong denotes ordinariness, universality, and harmony (Chiu, 2000; Kim et al., 2006). Individuals with high Zhongyong thinking can think of others as well as themselves (Yang et al., 2016) and tend to stand in the middle of the LMX rank. Their positive or unfavorable comparisons to others on LMXs become less prominent, hence diminishing the positive association between LMXSC and self-esteem.

Overall, our research has several important theoretical implications for the existing literature. First, we provide an integrated theoretical framework to unfold the effects of LMXSC on job performance and OCB by drawing from the social exchange and social comparison theories. Second, it represents a first step towards comprehending the roles of guanxi and Zhongyong thinking in the relationship between LMXSC and job performance and OCB in the Chinese cultural context. Such an examination largely enhances our knowledge of LMXSC in the Chinese context. Third, by demonstrating that Zhongyong thinking adversely affects the association between LMXSC and self-esteem, we are able to recognize that LMXSC is not always effective for all employees, and even superior LMX over their colleagues may become a burden for certain employees.

Next, this article is structured into three major sections. First, the present study expands on a clear theoretical framework based on social exchange theory and social comparison theory to hypothesize how and when LMXSC impacts employee job performance and OCB along two paths. Second, the present study uses a multi-source and multi-time field study to evaluate our theoretical model in the Chinese workplace. Third, theoretical and practical implications of LMXSC are discussed.

## 2. Theory and hypothesis

### 2.1. Social exchange path: LMXSC, perceived obligation, job performance, and OCB

Social exchange theory is a typical theoretical paradigm for explaining supervisor-subordinate interaction (Kim S. L. et al., 2017). The central tenet of social exchange theory is that resources are transferred through a reciprocal process in which one party wishes to return (an eye for an eye) the positive (or negative) behavior of another party (Gergen, 1969). In addition, the quality of the reciprocal process is largely determined by the relationship between the giver and the receiver (Blau, 1964). In a team, leaders differentiate in their treatment of followers, resulting in LMXs ranging from low to high between the leader and each employee (Sparrowe and Liden, 1997). Low-quality LMX relationships encompass mostly economic exchanges, which means formal, role-defined interactions and contractual exchanges between leaders and followers, whereas high-quality LMX relationships are based on social exchanges, which are typified by liking, trust, support, and respect (Cogliser et al., 2009). In general, employees with high LMX tend to respond to the preferential treatment received from the leader by meeting the leader’s expectations or increasing the leader’s interest, a process known as reciprocation (Uhl-Bien and Maslyn, 2003). However, a good-quality LMX relationship in the context of other high-quality relationships erodes its significance and may become inadequate to elicit strong feelings of obligation.

As previously mentioned, LMXSC depicts an employee’s subjective judgments of their own relative LMX position in comparison to their coworkers (Choi et al., 2020); a high level of LMXSC indicates that the employee has a better LMX connection with the leader than coworkers. LMXSC denotes a very valued connection, suggesting that the leader will commit more resources to this relationship than to others (Lee et al., 2019). That is, LMXSC can help to recognize the surrounding context of an employee’s LMX, where multiple LMX relationships exist between a leader and their followers, and determine to whether or not his or her LMX holds value (Pan et al., 2021). If followers with a high LMXSC have become the leader’s favorites, meaning they have received more instrumental resources and support from their leaders, such as information, learning opportunities, good evaluations, or promotion, than their coworkers (Thomas et al., 2013). In addition, they also get more affective support, like, and trust from their leader than other coworkers (Marescaux et al., 2021). According to social exchange theory, scarcity, such as when you have something that most others do not, enhances the value of any deal (Blau, 1964). Clearly, a high LMXSC consists of a variety of resources that each employee would deem important and aspire to obtain in their organization. In this case, according to the logic of social exchange, employees with a high LMXSC would feel more obligated to respond to their leaders because they know that their leaders treat them better than other coworkers, giving them access to vital and exclusive resources (Singh and Vidyarthi, 2018). Lee et al. (2019) found that LMXSC is positively related to felt obligation.

Moreover, when employees feel obligated to their leaders, they will demonstrate positive work behaviors (Dulebohn et al., 2012) in order to satisfy their leaders’ expectations. Work performance is one of the most important factors determining the overall success of an organization; as a result, it has evolved into the primary focus of leaders (Rich et al., 2010). Work performance is increasingly seen as including concepts such as “job performance,” defined as outputs explicitly required by a job role, and “OCB,” defined as discretionary behaviors that advance the interests of organizations (Organ, 1988). We contend that there are two primary ways for employees to complete their duties in order to reciprocate their leader: job performance and OCB. Previous research has documented that employees would positively finish their job duties or work hard to fulfill their sense of obligation (Basit, 2017). Wong et al. (2022) found that employees’ feeling of obligation toward LMX leads to improvements in their job performance. Kim et al. (2010) found that LMX leads to a higher OCB. Overall, we propose that:

*H1*: Perceived obligation positively mediates the relationship between LMXSC and (a) job performance, and (b) OCB.

### 2.2. The moderating role of guanxi

As stated earlier, LMXSC instills in employees a feeling of obligation towards the leader. In this section, we investigate which situations LMXSC will have a greater or lesser effect on perceived obligation using the social exchange perspective. In particular, we argue that guanxi makes the link between LMXSC and perceived obligations stronger. Guanxi is the core concept for comprehending Chinese social structure and interpersonal interaction among Chinese, who are primarily relationship-oriented (Chen and Chen, 2004). That is, guanxi is widespread and all-encompassing in Chinese society and is seen as a major factor in influencing how Chinese interact with and treat others (Wang et al., 2005). The concept of guanxi originates from Confucianism, which has identified the five most fundamental relationships between people: monarch, father and son, couple, brother, and friend (Haibo, 2020), namely the “five lun.” Five lun desires that all parties in a particular relationship express and behave in accordance with their social roles. In Chinese companies, supervisor-subordinate guanxi is an informal and personal relationship characterized by personal contact, emotional engagement, and mutual support beyond the workplace (Zhang et al., 2016). Guanxi develops mostly *via* non-work-related social contacts, such as having dinner, giving gifts, and helping, and emphasizes the principle of communal sharing between parties (i.e., the development of significant personal obligations based on specific or emotional ties; Zhang et al., 2015). In contrast, LMX originates from work-related interactions that are confined to the workplace, include only work-related exchanges, and emphasize the equity-matching principle (i.e., the fair exchange between performance and rewards for leaders and employees; Martin et al., 2005). We argue that there are three reasons to explain why guanxi may be a potential moderator in the relationship between LMXSC and perceived obligation.

First, LMXSC is based on relationships and activities defined by formal, role-based interactions in the workplace, while guanxi is a sort of informal relationship that occurs in the personal lives of employees (Zhang et al., 2015). Employees who have a high LMXSC as well as guanxi with leaders are more likely to form social exchange relationships in their work interactions as well as personal close relationships that include various emotional support in the life domain (Han and Altman, 2009). In different domains, we argue that two key forms of the employees’ relationship ties—organizational ties to the leaders and life ties to the leaders—can create a large and rich social network for the employees and enable them to meet the leaders’ needs and goals to maintain these stable ties with leaders who provide them with important information, social support, and interesting assignments (Balkundi and Kilduff, 2006). A growing number of studies have shown that the more extensive and intimate a person’s social network, the more reciprocal obligations that individual embedded in the social network should fulfill (Lin and Lo, 2015).

Second, guanxi is founded on affection and a feeling of reciprocal responsibility (Thibaut and Kelley, 1959), emphasizing emotional commitment and a desire to look out for one another. Nonetheless, LMXSC, which is derived from LMX, incorporates social and economic exchanges, emphasizing formal role duties and a feeling of indebtedness (Lee et al., 2019). Zhang et al. (2016) proposed that LMX is limited in cognition-based support between two parties, which is mostly reliant on the calculation of give and return, and that two parties prefer to maintain a balance of give and take. Accordingly, LMXSC reflects that employees get better or worse LMXs compared with other coworkers (Weng et al., 2020). In contrast, guanxi is a parochial and emotive bond founded on a shared understanding between individuals (Li, 2007). Guanxi is affection-based support between two parties; when employees have strong guanxi with the leader, they can identify the leader’s desires when determining their future activities (Zhang et al., 2016). Thus, those high in guanxi and LMXSC feel a double (cognitive and emotional) obligation to repay their leader.

Third, the communal sharing principle makes people in guanxi perceive a greater obligation to reciprocate, although they would get fewer rewards and benefits (Miao et al., 2020). The equity matching principle requires employees in LMX or LMXSC to repay based on what they receive from leaders. Guanxi is clearly long-term oriented and is based on total commitment and trust (Aryee et al., 2002). More importantly, guanxi manifests itself as being other-oriented, and this other-oriented characteristic causes people to not only have enough understanding of and concern for the other’s interests but also to not care if they give more or get less (Chen and Chen, 2004). Thus, we propose:

*H2*: Guanxi positively moderates the relationship between LMXSC and perceived obligation.

### 2.3. Social comparison path: LMXSC, self-esteem, job performance and OCB

Social comparison theory proposes that social comparison is a basic aspect of human social existence (Buunk and Gibbons, 2007), that individuals frequently compare themselves to others in order to learn more about themselves, and that the outcomes of these comparisons influence their future behaviors. Employees have a natural tendency to compare themselves to peers who are repeatedly exposed same or comparable leaders, events, practices, and experiences (Kim et al., 2010). In the workplace, leaders tend to distinguish their treatment of their followers, which builds social exchange ties (high LMX) with certain ingroup followers and economic exchange ties (low LMX) with other outgroup followers (Henderson et al., 2009). This kind of LMX differentiation leads employees to participate in social comparisons with their coworkers in order to ascertain their LMX rank, and thus, Vidyarthi et al. (2010) refer to this process as LMXSC.

We argue that LMXSC evaluations are a significant factor in the development of self-esteem. Self-esteem, defined as the extent to which an individual believes he or she is competent, important, and worthy (Bandura, 1986), is a vital element of self-evaluation that influences individual behavior. Self-esteem can be derived through social comparisons that convey self-relevant information (Wheeler and Miyake, 1992). According to the direction of social comparison, it may be categorized as either upward comparison (i.e., comparing oneself to others who are better off; Festinger, 1954) or downward comparison (i.e., comparing with others who are worse off; Hakmiller, 1966). Furthermore, people who are in downward comparisons have a positive self-image, while people who are in upward comparisons have a negative self-image (Maslach, 1993). Similarly, employees with a high LMXSC prefer to engage in downward comparisons, and their dominant LMX position gives them a sense of self-worth and competence, resulting in a strong feeling of self-esteem. Conversely, employees with a low LMXSC tend to make upward comparisons, and their inferior LMX status causes them to believe they lack abilities, significance, and worth (Arain et al., 2017). In addition, the symbolic interactionist approach indicates that a person’s self-concept is rooted in interpersonal connections (Mead, 1934), and that our self-perceptions are significantly influenced by how others perceive and see us (e.g., the looking-glass self; Cooley, 1972). For example, Leary et al. (1995) argued that self-esteem level is the result of being liked or disliked by others. Ferris et al. (2015) also found that when employees are disliked by others, they will have a low level of self-esteem. A high LMXSC ranking indicates that the targeted employees are the leaders’ favorites and get special attention, trust, or favor, while a low LMXSC indicates the likelihood that a person has already been excluded from the support and attention of a leader. Overall, LMXSC may positively lead to self-esteem.

Moreover, we argue that self-esteem is positively expected to relate to job performance and OCB. As noted previously, employees with low self-esteem are more likely to see themselves as failures and underperform in comparison to others (Rosenberg, 1965), while employees with high self-esteem see themselves as capable and valuable. According to the self-verification theory, people act in line with their self-evaluation (Korman, 1976). Therefore, employees with a high degree of self-esteem should be more confident and motivated to demonstrate outstanding job performance and even participate in OCB. Research has consistently shown that there is a positive relationship between self-esteem and job performance (e.g., Strauss, 2005). Ma et al. (2021) found that employees with high self-esteem are more likely to participate in OCBs that go beyond formal job obligations, such as maintaining exceptional role performance and assisting others. However, low-self-esteem employees should be less likely to participate in job performance and OCB to demonstrate that they do not have enough abilities or much capability, or to demonstrate to others that they are failures (Avey et al., 2011). Thus, we propose that

*H3*: Self-esteem positively mediates the relationship between LMXSC and (a) job performance, and (b) OCB.

### 2.4. The moderating role of Zhongyong thinking

According to the social comparison theory, not everyone would experience the same consequences of social comparison when confronted with the same a comparable social comparison scenario (Lyubomirsky and Ross, 1997). Individual characteristics impact how individuals respond to the outcomes of social comparisons (Sedikides and Brewer, 2001). Specifically, White and Lehman (2005) claim that people from diverse cultural origins have distinct social comparison outcomes. In general, individuals from Eastern and Western cultural contexts vary in how they make sense of objects, people, events, and surroundings (Markus and Kitayama, 1991). According to Chang and Yang (2014), one of the fundamental ways that Chinese people think is through the lens of Zhongyong, which is concerned with the ways in which Chinese people see things, other people, and the world around them (Pierce and Aguinis, 2013). Chinese individuals prefer to avoid extreme perspectives on the environment, place themselves in the middle of any given ranking system, and make judgments and take actions in a moderate manner (Wei et al., 2020). Zhongyong maintains that an individual’s feelings, thoughts, and actions should never be experienced or expressed outside of the bounds of moderation, that is, neither in an excessive amount nor in an inadequate amount (Ji and Chan, 2017). Wu and Lin (2005) proposed that people with Zhongyong thinking can think in multiple dimensions, such as time, space, and roles, can recognize the dialectical relationship between contradictory elements (e.g., everything has both a dark and a light side); and can connect objects, people, and the environment from a holistic perspective.

Zhongyong thinking influences how employees view and respond to LMXSC. First, employees with high Zhongyong thinking tend to be more perspective-taking and see the world from others’ viewpoints (Wei and Wang, 2020). In this case, high-level LMXSC members who make downward comparisons may consider and share the thoughts and sensations of their less fortunate coworkers, thereby weakening their sense of relative superiority (Yu and Yang, 2022). Second, employees with Zhongyong thinking might analyze LMXSC from a long-term vantage point (Chou et al., 2014), indicating that they are less prone to be misled by the relatively high LMX standing at present. In fact, the present high LMXSC is not fixed in stone, and the current high LMXSC does not guarantee that it will be maintained in the future. When employees with Zhongyong thinking are able to recognize this point, the benefits associated with having a high LMXSC in the social comparison process are diminished for those employees. As a result, possessing a high LMXSC ranking could no longer serve as a motivating factor for those employees to develop their self-esteem. Conversely, when low LMXSC members with high Zhongyong thinking engage in upward comparisons, they may put themselves in the shoes of better-off coworkers and think that their status level (i.e., high LMXSC) is attainable for them in the future (Buunk and Ybema, 1997). Third, the connotation of integration in Zhongyong thinking motivates employees to develop cooperative beliefs rather than competitive beliefs (He and Li, 2021). A competitive belief makes employees more likely to feel alienated from the team, leading them to care more about the outcomes of social comparison, thereby highlighting the effects of LMXSC (Garcia et al., 2013). That is, competitive belief emphasizes the contrast effects of social comparison, making LMXSC more important in the process of developing individual self-esteem (Morse and Gergen, 1970). Cooperative mindset causes employees to diminish the disparities between themselves and their low LMXSC coworkers and to view their superior ranks in terms of LMXs as common and reachable by their coworkers, thereby lowering their self-esteem (Buunk et al., 1990). Finally, Zhongyong thinking personnel would understand that an excess of any good thing is ultimately undesirable (Pierce and Aguinis, 2013). Zhongyong thinking highlights universal advocacy for proportionality over extremity (Wei et al., 2020). In such a context, low LMXSC members with high Zhongyong thinking might perceive the current low LMXSC as no bad thing, while high LMXSC members with high Zhongyong thinking might perceive the current high LMXSC as no good thing. Thus, the positive effect of LMXSC on self-esteem is diminished by Zhongyong thinking. Overall, we propose our hypothesis:

*H4*: Zhongyong thinking negatively moderates the relationship between LMXSC and self-esteem.

## 3. Methods

### 3.1. Participants and procedure

China is now the biggest domestic tourism market, the largest international tourism consumer, and the fourth largest tourist destination in the world. The hotel industry is an essential pillar sector in China and plays an important role in the national economy’s growth. Although research efforts have been more focused on the impacts of LMXSC, the question of whether LMXSC is beneficial to the hotel business remains unanswered. The hotel sector, which is characterized by interpersonal interaction, is an appropriate setting for analyzing the comparison of interpersonal relationships such as LMXSC. Therefore, the context of our current investigation focuses on the Chinese hotel industry.

We collected multisource data across three time points from 10 hotel organizations located in China. We obtained a strong endorsement of our work from the human resources departments of these organizations. This study project included employees from different departments, and we made sure to ask them if they were interested in doing our survey before inviting them. We provided all participants with a thorough explanation of how we protected their anonymity by never disclosing their personal information in exchange for their voluntary participation. We used identifying numbers to match the survey answers of employees and their supervisors across all three waves.

Each of the three waves was separated by 2 weeks. During Wave 1, questionnaires were administered to 478 subordinates. Respondents were asked to provide demographic information and their perceptions about LMXSC, LMX, guanxi, and Zhongyong thinking. A total of 446 usable responses were obtained, representing a response rate of 93.31%. Two weeks later, we delivered Wave 2 surveys to 446 employees, asking them to rate their perceived obligation and self-esteem, and we received 415 valid replies, for a response rate of 93.05%. Two weeks after Wave 2, we distributed Wave 3 questionnaires to supervisors (who supervised the 415 subordinates) and asked them to rate the work performance and OCB of their subordinates. 370 valid responses were identified, resulting in a response rate of 89.16%. Finally, we got 370 dyads of data.

The employee sample was 60% female; employees averaged 29.04 years of age (*SD* = 6.56) and reported working an average of 53.24 months (*SD* = 60.73) in their organizations. In terms of marital status, 61.4% of them were married. In terms of education, the sample included those with a junior high school diploma or less (3.2%), technical secondary school (11.6%), high school (6.2%), junior college (31.6%), a bachelor’s degree (42.4%), and a postgraduate degree (4.2%).

### 3.2. Measures

Unless otherwise specified, all measures were scored using a Likert scale ranging from 1 (strongly disagree) to 7 (strongly agree). In order to convert English scales to Chinese scales, translation and back-translation were utilized (Brislin, 1980).

#### 3.2.1. LMXSC

Employees were assessed for LMXSC with Vidyarthi et al. (2016) 6-item measure. Included in the sample items was “I have a better relationship with my manager than most others in my work group” (Cronbach’s alpha = 0.938).

#### 3.2.2. Perceived obligation

Perceived obligation was measured using the seven-item scale created by Eisenberger et al. (2001). An example item was, “I feel a personal obligation to do whatever I can to help my leader achieve his/her goals” (Cronbach’s alpha = 0.940).

#### 3.2.3. Self-esteem

Rosenberg (1965) 10-item self-esteem scale was used. Sample items include, “On the whole, I am satisfied with myself” (Cronbach’s alpha = 0.939).

#### 3.2.4. Guanxi

Guanxi was used with six items developed by Law et al. (2000). Sample items include, “I always actively share with my supervisor about my thoughts, problems, needs and feelings” (Cronbach’s alpha = 0.894).

#### 3.2.5. Zhongyong thinking

Zhongyong thinking was measured using 13 items developed by Taiwanese scholars Wu and Lin (2005). The measurement used a 5-point Likert-type scale (1 = strongly disagree, 5 = strongly agree) to rate Zhongyong thinking. An example item was, “I will take into account the conflicting views from each other in discussion” (Cronbach’s alpha = 0.948).

#### 3.2.6. Job performance

Because our samples are from hotels and we utilize service performance to gauge their job performance. Job performance was assessed using a seven-item measure by Liao and Chuang (2004). An example item was, “being friendly and helpful to customers” (Cronbach’s alpha = 0.901).

#### 3.2.7. OCB

OCB was assessed using a fourteen-item developed by Williams and Anderson (1991). Sample items include, “I help others who have heavy workloads” and “I conserve and protects organizational property” (Cronbach’s alpha = 0.953).

#### 3.2.8. Control variables

We controlled for followers’ gender (0 = male; 1 = female), age (in years), organizational tenure (in months), marital status (1 = single; 2 = married), and education (1 = junior high school or below; 2 = technical secondary school; 3 = high school; 4 = junior college; 5 = bachelor’s degree; 6 = master’s degree or above) because employees’ demographic variables may impact their attitudes as well as their performance (Lam et al., 2015). We also controlled for LMX because it is one of the most closely related constructs to LMXSC. We measured LMX with a 10-item scale developed by Liden et al. (1993). Sample items are “I know where I stand with my supervisor” and “My supervisor recognizes my potential” (Cronbach’s alpha = 0.902).

## 4. Results

### 4.1. Descriptive statistics

The mean, standard deviation, and correlations among our variables are shown in Table 2. LMXSC is positively related to perceived obligation (*r* = 0.255, *p* < 0.01), self-esteem (*r* = 0.254, *p* < 0.01), job performance (*r* = 0.324, *p* < 0.01), and OCB (*r* = 0.184, *p* < 0.01). Perceived obligation is positively related to job performance (*r* = 0.270, *p* < 0.01), and OCB (*r* = 0.340, *p* < 0.01). Self-esteem is positively related to job performance (*r* = 0.239, *p* < 0.01), and OCB (*r* = 0.261, *p* < 0.01).

**Table 2:** Means, standard deviations, and bivariate correlations among studied variables.

Variable	1	2	3	4	5	6	7	8	9	10	11	12	13
Sex													
Age	0.109*												
MS	0.236**	0.552**											
OT	0.071	0.702**	0.355**										
Education	0.075	−0.114*	0.054	−0.003									
LMXSC	0.053	0.103*	0.080	0.041	0.008								
LMX	−0.099	0.081	−0.083	−0.015	−0.131*	0.280**							
PO	−0.002	0.026	0.003	−0.054	0.092	0.255**	0.036						
SE	−0.004	0.052	0.088	0.016	0.091	0.254**	−0.013	0.278**					
Guanxi	0.063	0.083	0.017	0.079	−0.065	0.027	−0.042	−0.036	0.073				
ZY	−0.042	−0.018	0.029	−0.099	−0.079	−0.062	0.007	−0.040	−0.063	−0.081			
JP	−0.070	0.007	−0.079	0.004	0.018	0.324**	0.008	0.270**	0.239**	0.033	−0.069		
OCB	0.024	0.078	0.039	0.067	0.054	0.184**	0.037	0.340**	0.261**	−0.020	−0.025	0.192**	
*Mean*	0.60	29.04	1.61	53.24	4.13	4.360	5.117	5.044	4.625	4.099	3.642	5.299	5.635
*SD*	0.491	6.558	0.488	60.725	1.194	1.261	1.112	1.138	0.982	1.322	0.848	1.107	1.045

N = 370. *p < 0.05. **p < 0.01. MS = marital status; OT = organizational tenure; PO = perceived obligation; SE = self-esteem; ZY = Zhongyong thinking, JP = job performance.

### 4.2. Common method bias

Harman’s one-factor test was used to test the common method bias (Chang et al., 2020). The results showed that the largest, extracted component accounts for only 20.386% of the total variance. In addition, confirmatory factor analyses were conducted. Fit indices were compared between two measurement models: (1) a model with a common method factor and (2) a model without a common method factor. The fit indices did not improve significantly (ΔCFI = 0.000, ΔTLI = 0.001, ΔRMSEA = 0.000 Δ SRMR = 0.005, respectively). Overall, these findings demonstrated that the likelihood of common method bias is, in fact, low.

### 4.3. Confirmatory factor analyses

We conducted a set of confirmatory factor analyses to examine whether our measures (i.e., LMXSC, perceived obligation, self-esteem, guanxi, Zhongyong thinking, job performance and OCB) captured distinctive constructs (see Table 3). Results suggested that the theorized seven-factor model [χ^2^(1864) = 3341.931, CFI = 0.919, TLI = 0.915, RMSEA = 0.046, SRMR = 0.044] fit the data better than six alternative models, demonstrating that these variables were distinct.

**Table 3:** Confirmatory factor analyses.

Model	Factors	χ2	df	Δχ^2^	RMSEA	CFI	TLI	SRMR
Model1	Seven-factor	3341.931	1864		0.046	0.919	0.915	0.044
Model2	Six-factor (LMXSC+PO)	5160.960	1870	1819.029**	0.069	0.820	0.812	0.077
Model3	Five-factor (LMXSC+PO + SE)	6805.879	1875	3463.948**	0.084	0.731	0.720	0.100
Model4	Four-factor (LMXSC+PO + SE + OCB)	9007.643	1879	5665.712**	0.101	0.611	0.595	0.127
Model5	Three-factor (LMXSC+PO + SE + OCB + JP)	10694.513	1882	7352.582**	0.112	0.519	0.501	0.136
Model6	Two-factor (LMXSC+PO + SE + OCB + JP + ZY)	13316.152	1884	9974.221**	0.128	0.376	0.353	0.176
Model7	One-factor	14559.309	1885	11217.378**	0.135	0.308	0.283	0.183

N = 370. **p < 0.01. PO = perceived obligation; SE = self-esteem; JP = job performance; ZY = Zhongyong thinking.

### 4.4. Tests of the hypotheses

We used the PROCESS macro (Model 4) to test the mediating effects of perceived obligation and self-esteem. Table 4 revealed that, after accounting for all control variables, LMXSC had a significant and positive effect on perceived obligation (*b* = 0.237, *p* < 0.01), self-esteem (*b* = 0.212, *p* < 0.01) and job performance (*b* = 0.251, *p* < 0.01). Perceived obligation had a significant and positive effect on job performance (*b* = 0.161, *p* < 0.01) and OCB (*b* = 0.257, *p* < 0.01). Self-esteem had a significant and positive effect on job performance (*b* = 0.145, *p* < 0.05) and OCB (*b* = 0.177, *p* < 0.01). In addition, results revealed that the indirect effect of LMXSC on job performance *via* perceived obligation was significant, estimate = 0.038, 95% CI = [0.008, 0.087]. Likewise, the indirect effect of LMXSC on OCB *via* perceived obligation was significant, estimate = 0.061, 95% CI = [023, 0.116]. Thus, H1a and H1b were supported. The indirect effect of LMXSC on job performance *via* self-esteem was significant, estimate = 0.031, 95% CI = [0.002, 0.072]. Likewise, the indirect effect of LMXSC on OCB *via* self-esteem was significant, estimate = 0.037, 95% CI = [0.010, 0.087]. Thus, H3a and H3b were supported.

**Table 4:** Results for estimated coefficients of the mediation model.

Variables	Mediator: perceived obligation			Mediator: self-esteem			DV: job performance			DV: OCB		
	b	SE	t	b	SE	t	b	SE	t	b	SE	t
Constant	3.442**	0.488	7.051	3.508**	0.422	8.320	3.613**	0.507	7.120	3.084**	0.487	6.331
Age	0.027	0.014	1.861	0.007	0.012	0.549	0.007	0.013	0.523	0.002	0.013	0.168
Sex	−0.050	0.121	−0.411	−0.097	0.104	−0.929	−0.153	0.111	−1.370	0.044	0.107	0.409
Marital status	−0.124	0.147	−0.944	0.115	0.127	0.904	−0.308*	0.136	−2.268	−0.042	0.130	−0.321
Organizational tenure	−0.003*	0.001	−2.123	−0.001	0.001	−0.044	0.000	0.001	0.325	0.001	0.001	1.062
Education	0.101*	0.049	2.047	0.069	0.043	0.083	−0.007	0.046	−0.143	0.014	0.044	0.309
LMX	−0.046	0.055	−0.841	−0.073	0.048	−0.083	−0.097	0.051	−1.906	0.014	0.049	0.279
LMXSC	0.237**	0.048	4.979	0.212**	0.041	0.272	0.251**	0.046	5.408	0.051	0.045	1.152
Perceived obligation							0.161**	0.050	3.249	0.257**	0.048	5.387
Self-esteem							0.145*	0.057	2.516	0.177**	0.055	3.203
*R* ^2^	0.087			0.085			0.183			0.157		
*F*	4.942**			4.781**			8.972**			7.438**		

Unstandardized regression coefficients are reported. *p < 0.05. **p < 0.01.

**Figure 1:**
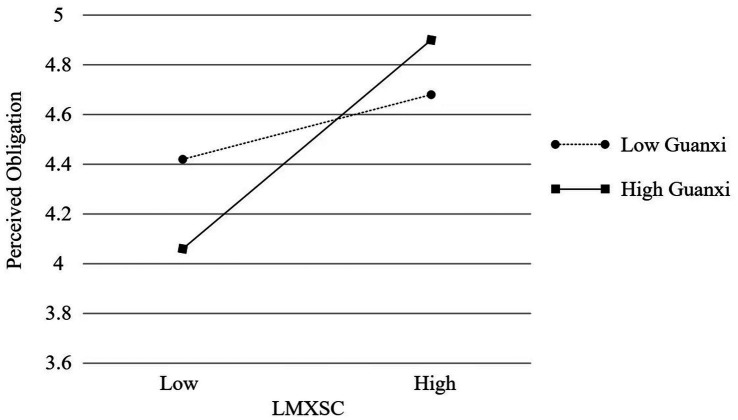
Interactive effect of LMXSC and guanxi on perceived obligation.

**Figure 2:**
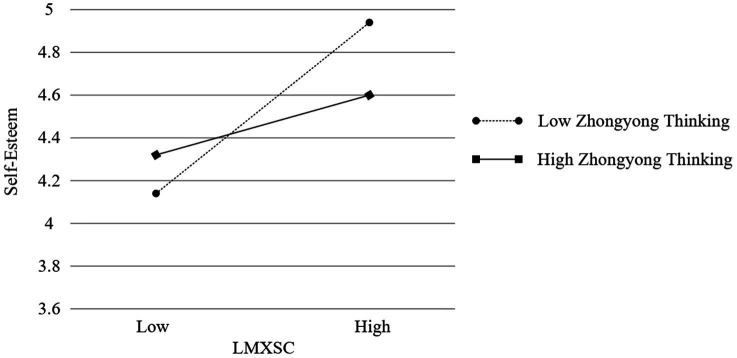
Interactive effect of LMXSC and Zhongyong thinking on self-esteem.

In addition, we tested the moderated mediation model using the PROCESS macro (Model 7). Table 5 revealed that, after accounting for all control variables, the interaction of LMXSC and guanxi was significant and positive in predicting perceived obligation (*b* = 0.146, *p* < 0.01). Simple slope tests indicated that the effect of LMXSC on perceived obligation was stronger at higher levels of guanxi (+1 *SD*; *b* = 0.448, *t* = 5.638, *p* < 0.01) than at lower levels (−1 *SD*; *b* = 0.156, *t* = 1.985, *p* < 0.05; Figure 1). Thus, Hypothesis 2 was supported. The interaction of LMXSC and Zhongyong thinking was significant and negative in predicting self-esteem (*b* = −0.113, *p* < 0.05). The effect of LMXSC on self-esteem was stronger at lower levels of Zhongyong thinking (−1 *SD*; *b* = 0.377, *t* = 5.421, *p* < 0.01) than at higher levels (+1 *SD*; *b* = 0.150, *t* = 2.153, *p* < 0.05; Figure 2). Thus, Hypothesis 4 was supported.

**Table 5:** Results for estimated coefficients of the moderated mediation model.

Variables	Mediator: perceived obligation			Mediator: self-esteem			DV: job performance			DV: OCB		
	b	SE	t	b	SE	t	b	SE	t	b	SE	t
Constant	4.517**	0.491	9.191	4.491**	0.426	10.542	4.688**	0.546	8.580	3.293**	0.530	6.214
Age	0.024	0.014	1.720	0.007	0.012	0.593	0.009	0.013	0.683	0.003	0.013	0.236
Sex	−0.030	0.120	−0.247	−0.088	0.104	−0.852	−0.169	0.111	−1.520	0.045	0.108	0.415
Marital status	−0.124	0.146	−0.848	0.105	0.126	0.832	−0.304*	0.135	−2.248	−0.048	0.131	−0.365
Organizational tenure	−0.003*	0.001	−2.004	−0.001	0.001	−0.773	0.000	0.001	0.146	0.001	0.001	1.062
Education	0.102*	0.049	2.075	0.063	0.042	1.479	−0.012	0.046	−0.271	0.011	0.044	0.247
LMX	−0.048	0.055	−0.878	−0.081	0.047	−1.696	−0.100	0.051	−1.957	0.011	0.049	0.232
LMXSC	0.302**	0.060	5.071	0.263**	0.052	5.098	0.311**	0.058	5.332	0.065	0.057	1.147
Guanxi	−0.047	0.057	−0.823				0.025	0.049	0.465	−0.029	0.052	−0.569
LMXSC*Guanxi	0.146**	0.052	2.802				−0.136**	0.050	−2.773	−0.041	0.047	−0.866
Perceived obligation							0.185**		3.683	0.262**	0.049	5.395
Zhongyong thinking				−0.038	0.050	−0.757	−0.036	0.053	−0.667	0.009	0.052	0.176
LMXSC*Zhongyong thinking				−0.113*	0.047	−2.429	−0.038	0.050	−0.756	−0.003	0.049	−0.070
Self-esteem							0.122*	0.058	2.104	0.175**	0.056	3.114
*R* ^2^	0.108			0.102			0.203			0.160		
*F*	4.845**			4.524**			6.954**			5.199**		

*p < 0.05. **p < 0.01.

**Figure 3:**
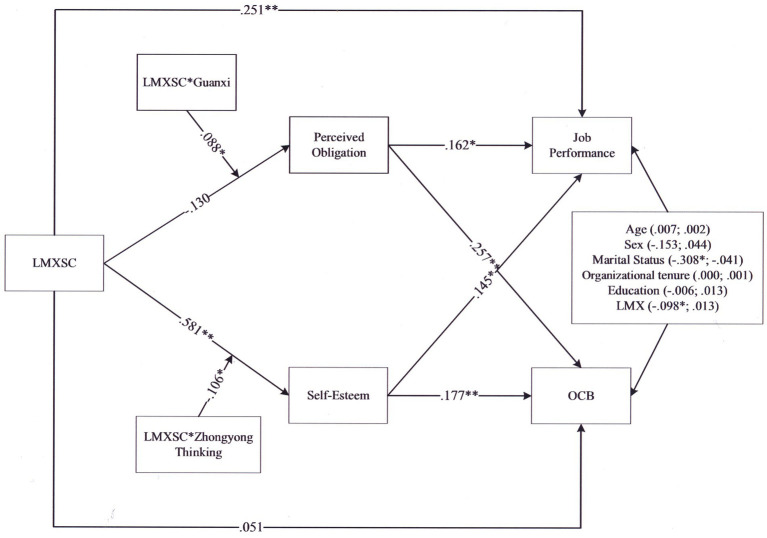
Hypothesized model using structural equation modeling. * *p* < 0.05, ** *p* < 0.01.

Table 6, for job performance, revealed that the index of moderated mediation when the mediator is perceived obligation and the moderator is guanxi was significant (index = 0.017; 95% CI = [0.003, 0.043]). The indirect effect of LMXSC on job performance *via* perceived obligation was significant and positive when guanxi was higher (+1 *SD*; estimate = 0.067; 95% CI = [0.020, 0.140]) but not when guanxi was lower (−1 *SD*; estimate = 0.023; 95% CI = [−0.001, 0.075]). For OCB, revealed that the index of moderated mediation when the mediator is perceived obligation and the moderator is guanxi was significant (index = 0.025; 95% CI = [0.004, 0.055]). The indirect effect of LMXSC on OCB *via* perceived obligation was significant and positive when guanxi was higher (+1 *SD*; estimate = 0.103; 95% CI = [0.046, 0.182]) but not when guanxi was lower (−1 *SD*; estimate = 0.036; 95% CI = [−0.004, 0.097]). For job performance, revealed that the index of moderated mediation when the mediator is self-esteem and the moderator is Zhongyong thinking was significant (index = −0.020; 95% CI = [−0.054, −0.001]). The indirect effect of LMXSC on job performance *via* self-esteem was significant and positive when Zhongyong thinking was higher (+1 *SD*; estimate = 0.022; 95% CI = [0.000, 0.066]) and lower (−1 *SD*; estimate = 0.055; 95% CI = [0.012, 0.115]). For OCB, revealed that the index of moderated mediation when the mediator is self-esteem and the moderator is Zhongyong thinking was significant (index = −0.026; 95% CI = [−0.066, −0.002]). The indirect effect of LMXSC on OCB *via* self-esteem was significant and positive when Zhongyong thinking was lower (−1 *SD*; estimate = 0.072; 95% CI = [027, 0.143]) but not when Zhongyong thinking was higher (+1 *SD*; estimate = 0.029; 95% CI = [−0.001, 0.078]). In addition, to perform a robust test, we utilized Mplus to undertake structural equation modeling in order to test all hypotheses; the results are shown in Figure 3. All hypotheses were also supported.

**Table 6:** Summary of indirect effects and conditional indirect effects.

Paths and effects	Estimates	SE	95% confidence intervals
*LMXSC-perceived obligation-job performance*			
Simple indirect effect	0.038	0.020	0.008, 0.087
Moderated mediation			
Lower guanxi (−1 SD)	0.023	0.019	−0.001, 0.075
Higher guanxi (+1 SD)	0.067	0.029	0.020, 0.140
Index of moderated mediation	0.017	0.010	0.003, 0.043
*LMXSC-perceived obligation-OCB*			
Simple indirect effect	0.061	0.023	0.023, 0.116
Moderated mediation			
Lower guanxi (−1 SD)	0.036	0.026	−0.004, 0.097
Higher guanxi (+1 SD)	0.103	0.035	0.046, 0.182
Index of moderated mediation	0.025	0.013	0.004, 0.055
*LMXSC-self-esteem-job performance*			
Simple indirect effect	0.031	0.018	0.002, 0.072
Moderated mediation			
Lower Zhongyong thinking (−1 SD)	0.055	0.026	0.012, 0.115
Higher Zhongyong thinking (+1 SD)	0.022	0.016	0.000, 0.066
Index of moderated mediation	−0.020	0.013	−0.054, −0.001
*LMXSC-self-esteem-OCB*			
Simple indirect effect	0.037	0.019	0.010, 0.087
Moderated mediation			
Lower Zhongyong thinking (−1 SD)	0.072	0.029	0.027, 0.143
Higher Zhongyong thinking (+1 SD)	0.029	0.020	−0.001, 0.078
Index of moderated mediation	−0.026	0.016	−0.066, −0.002

## 5. Discussion

Integrating social exchange theory and social comparison theory, we tested an integrated model to link LMXSC with employee job performance and OCB in the Chinese context. This research helps us deeply understand how and when LMXSC affects employee job performance and OCB in a particular Chinese cultural setting. We found that LMXSC indirectly impacts job performance and OCB *via* perceived obligation and self-esteem. Furthermore, we presented one contextual cultural factor (i.e., guanxi) as a boundary condition for the effects of LMXSC on perceived obligation and one individual cultural factor (i.e., Zhongyong thinking) as a boundary condition for the effects of LMXSC on self-esteem.

### 5.1. Theoretical implications

Our research provides several contributions to the existing literature. First, despite some empirical studies on the implications of LMXSC (Vidyarthi et al., 2010; Lee et al., 2019), our theoretical knowledge of this concept remains restricted and incomplete. The mechanisms that transmit the effects of LMXSC on job performance and OCB have not been systematically integrated. This study examines two pathways from LMXSC to work performance and OCB: one through perceived obligation and the other *via* self-esteem, which gives a rather thorough picture of how the effects of LMXSC manifest. Social exchange and social comparison seem to be equally plausible pathways for LMXSC, according to the LMXSC literature (Tse et al., 2018; Lapointe et al., 2020; Weng et al., 2020). However, Vidyarthi et al. (2016), Sharma et al. (2020), and Lee et al. (2019) address the processes behind the effects of LMXSC from only one theoretical approach, which largely limits the understanding of various mechanisms underlying the benefits associated with LMXSC. In this research, we adopted social exchange theory and social comparison theory simultaneously and found that perceived obligation and self-esteem concurrently mediate the relationship between LMXSC and job performance and OCB. That is, by assessing these two functions of LMXSC, we were able to discover social exchange and social comparison processes in the form of perceived obligation and self-esteem, respectively, which translated LMXSC into job performance and OCB. In doing so, we provide clear knowledge that the outcomes of the LMXSC are produced by two different underlying psychological processes.

Second, compared to earlier studies, the most notable aspect of this study is the cultural situations that influence the effects of LMXSC. Although Tse et al. (2013), Weng et al. (2020), Liu et al. (2022), and Huang et al. (2015) used Chinese samples to examine the generalizability of the LMXSC theory, they did not take into account how and when specific Chinese cultural factors influence the outcomes of LMXSC. Chinese culture, based on Confucian culture, is obviously different from Western culture (Hill, 2006). Regarding interpersonal interaction, guanxi and Zhongyong thinking are two distinctive and unique elements in Cunfucian culture (Ma et al., 2018). Guanxi includes informal, non-work relationships between employees and supervisors, while LMXSC and LMX are based on official, work relationships (Zhang et al., 2015); there may be interaction effects between these two forms of relationships. Zhongyong thinking determines how Chinese individuals interact with others and evaluate or make sense of their surroundings, which may affect how they respond to their upward and downward comparisons, especially their relatively high or low LMX ranks in comparison to their peers. According to the social exchange perspective, guanxi facilitates social exchange by fostering a strong feeling of mutual benefit (Warren et al., 2004), and thus we found that guanxi positively moderated the relationship between LMXSC and perceived obligation. According to the social comparison perspective, we found Zhongyong thinking, as a common way of thinking adopted by Chinese people (Zhou et al., 2021), negatively moderates the relationship between LMXSC and self-esteem. That is, if employees have a high level of Zhongyong thinking, the positive effects of LMXSC on self-esteem are largely diminished. Our work is the first to analyze the cultural influences on this connection, and it reflects previous demands for consideration of individual cultural values and contextual cultural elements. Our research facilitates LMXSC scholars’ consideration of cultural aspects that may increase or decrease the impact of LMXSC understandings and helps explain distinctive management circumstances in a Confucian society, thereby contributing significantly to indigenous organizational studies on LMXSC in high-context nations.

Third, by investigating how guanxi moderates the link between LMXSC and perceived obligation, we add to a fuller knowledge of social exchange theory. LMXSC derives from the official and work-related LMX, while guanxi is a private and informal connection. Combining these two distinct forms of relationships between leaders and their followers, we investigate how LMXSC interact with guanxi and the effects of these interactions on perceived obligation based on the social exchange theory. We found that increased guanxi strengthens the positive link between LMXSC and perceived obligation due to the need of reciprocity in both relationships. To our knowledge, this is the first research to analyze the interplay between LMXSC and guanxi, thus expanding our grasp of social exchange theory, which illuminating the nature of distinct relationships and their accompanying obligation requirements.

Fourth, by demonstrating that the link between LMXSC and self-esteem is moderated by Zhongyong thinking, our study contributes to a deeper understanding of this relationship. Previous research tends to presume that the social comparison conclusions of LMXSC necessarily result in favorable outcomes, while disregarding the question of who, in particular, would have positive outcomes (Lee et al., 2019). We examined the effects of LMXSC in a Chinese social context by investigating Zhongyong thinking as a moderator that influences the relationships among LMXSC, self-esteem, job performance, and OCB. We found that LMXSC has a greater positive effect on self-esteem when Zhongyong thinking is low rather than high. This is because employees with Zhongyong thinking compare themselves to other coworkers who are in a low LMXSC situation, recognize that their high LMXSC does not guarantee that they will always be in a relatively high LMX position, and believe that everything, including LMXSC, may have both positive and negative aspects. After considering the aforementioned considerations, they find high LMXSC less appealing, and the benefits of high LMXSC are significantly lessened. Thus, when Zhongyong thinking is high, the positive effects of high LMXSC on self-esteem are weakened, which in turn limits employees’ job performance and OCB. This conclusion prompts us to consider if being high LMXSC is always advantageous for employees, which extend our existing understanding of LMXSC deeply. In addition, we observed that Chinese cultural factors have mixed impacts on LMXSC by exhibiting the positive moderator of guanxi in the link between LMXSC and perceived obligation and the negative moderator of Zhongyong thinking in the relationship between LMXSC and self-esteem. This finding sheds even more insight on the complicated nature of LMXSC across various cultures.

Finally, since the outbreak of the COVID-19 pandemic, over 3 years have passed. The COVID-19 epidemic has thrust mankind into an uncharted period marked by anxiety and helplessness (Trougakos et al., 2020). During these 3 years, employees around the globe endured extreme psychological stress, fear, and anxiety, which directly and indirectly hampered their various performance, including job performance and OCB (Kumar et al., 2021; Lee et al., 2021; Pfeifer et al., 2021). Based on social exchange theory and social comparison theory, we found that LMXSC positively improves job performance and OCB *via* perceived obligation and self-esteem, which may provide an insight into how employees in the context of the COVID-19 pandemic keep and increase their performance.

### 5.2. Practical implications

Based on our findings, our research has several practical implications for organizations. First, we found that LMXSC had a positive effect on perceived obligation and self-esteem, which in turn improved employee job performance and OCB. Thus, managers should acknowledge that employees are concerned with their relative LMX position in the workplace (Epitropaki et al., 2016), and thus they can build distinct work connections with their employees and treat them differently depending on the quality of their work relationships. In especially for important or talented employees, managers should let them perceive high LMXSC in order that they have a high perceived obligation and self-esteem to enhance their performance outcomes through various means. For instance, leaders might tell these employees vocally that they are their favorites, which conveys the signal of their LMX rank.

Second, we found that guanxi positively moderated the relationship between LMXSC and perceived obligation. Thus, managers can use the means of guanxi to influence the positive effects of LMXSC and perceived obligation. For example, if a manager wishes to inspire an employee with a low LMXSC, he or she might cultivate guanxi with this employee in order to increase his or her sense of obligation, job performance, and OCB. Leaders can share their thoughts, opinions, and emotions regarding work and life with employees, assist employees in resolving their life problems, call or connect *via* social media apps or visit in person, participate in social activities with employees, such as having dinner or enjoying entertainment, and become acquainted with the families of employees. All of these tactics may help leaders proactively build guanxi with their employees.

Third, we found that Zhongyong thinking adversely moderates the positive relationship between LMXSC and self-esteem; hence, managers must recognize the significance of Zhongyong thinking in the relationship. In short, Zhongyong thinking helps employees with low LMXSC while harming employees with high LMXSC. Thus, managers should communicate with their employees to identify the level of LMXSC perceived by them, know if their employees with high LMXSC have Zhongyong thinking, and encourage their employees with low LMXSC to make sense their standings with Zhongyong thinking.

### 5.3. Strengths, limitations, and future research directions

The current research has some strengths. For example, we use the temporal separation of focal variables and multi-source data (leaders and employees) to decrease the common method bias. However, our research has several limitations. First, employees simultaneously provided ratings on LMXSC, perceived obligation, and self-esteem, which may raise concerns about the common method bias. In addition, the subjective bias of leaders might influence how they rate job performance and OCB. Thus, further research can use objective performance metrics (e.g., salary) or multiple peer-rated OCBs to test our model. Second, we only examine the influence of guanxi and Zhongyong thinking on the LMXSC function in Chinese culture. No doubt, Chinese and Western cultures distinguish individuals in several ways. For instance, Chinese culture causes Chinese to have an interdependent self, to be collectivist, and to have a greater power distance, while western culture causes westerners to have a dependent self, to be individualist, and to have a smaller power distance (Bochner and Hesketh, 1994; Jung and Avolio, 1999; Lee et al., 2000). Additional study may investigate if and how other Chinese cultural characteristics, such as interdependent self, collectivism, and power distance, impact the functions of LMXSC. Third, by detecting the negative moderator of Zhongyong thinking in the link between LMXSC and self-esteem, we recognize that high LMXSC is not always beneficial for all employees, hence revealing the possible negative side of LMXSC. Thus, further study should recognize that LMXSC may be a mixed blessing and investigate how and when high LMXSC may burden employees and impact their job performance and OCB. Finally, we only explore perceived obligation and self-esteem as mechanisms in the relationship between LMXSC and job performance and OCB. Further research can explore other variables, such as self-verification (Shantz and Booth, 2014), as an additional mediator based on other acceptable theories’ perspectives.

## 6. Conclusion

Little is known about how and when LMXSC influences employee job performance and OCB in the Chinese context. Using social exchange theory and social comparison theory, we explained the indirect effects of LMXSC on job performance and OCB *via* perceived obligation and self-esteem. In addition, the results highlight the critical role of guanxi and Zhongyong thinking in shaping perceived obligation, self-esteem, and subsequent employee effectiveness.

## Data availability statement

The raw data supporting the conclusions of this article will be made available by the authors, without undue reservation.

## Ethics statement

The studies involving human participants were reviewed and approved by School of Economics and Management, Yanshan University’s ethics committee. The patients/participants provided their written informed consent to participate in this study.

## Author contributions

CY and YC finished the manuscript. AC and SA collected the data and conducted the statistical analysis. All authors contributed to the article and approved the submitted version.

## Funding

This research was funded by the National Science Foundation of China (Grant no. 72172137) and the Chinese Ministry of Education (Grant no. 21YJA630103).

## Conflict of interest

The authors declare that the research was conducted in the absence of any commercial or financial relationships that could be construed as a potential conflict of interest.

## Publisher’s note

All claims expressed in this article are solely those of the authors and do not necessarily represent those of their affiliated organizations, or those of the publisher, the editors and the reviewers. Any product that may be evaluated in this article, or claim that may be made by its manufacturer, is not guaranteed or endorsed by the publisher.
